# Structural Insights into the Glycosylphosphatidylinositol Mannosyltransferase I Complex from *Candida glabrata*

**DOI:** 10.3390/jof11110819

**Published:** 2025-11-19

**Authors:** Hui Sun, Weihong Wu, Xiaomei Li, Yang Deng, Jiarong Huang, Meng Yin, Zhaofeng Yan

**Affiliations:** 1 School of Biomedical Sciences, Hunan University, Changsha 410082, China; 2 Shanxi Academy of Advanced Research and Innovation, Taiyuan 030032, China; 3 Hunan Provincial Key Laboratory of Anti-Resistance Microbial Drugs, The Third Hospital of Changsha, Changsha 410118, China

**Keywords:** *Candida glabrata*, GPI, mannosyltransferase, cryo-EM

## Abstract

The global rise in resistance to first-line antifungal agents highlights the urgent need for new therapeutic strategies. Glycosylphosphatidylinositol (GPI)-anchored protein biosynthesis is an attractive target. The GPI mannosyltransferase I (GPI-MT-I), composed of Gpi14 and Pbn1, catalyzes the essential first mannose transfer from dolichol-phosphomannose (Dol-P-Man) to the GPI precursor. This initial mannosylation is critical for fungal cell wall integrity, yet the molecular basis of GPI-MT-I assembly and substrate recognition remains poorly understood. Here, we present the cryo-EM structure of *Candida glabrata* GPI-MT-I in complex with Dol-P-Man, revealing how Gpi14 and Pbn1 form a stable complex and engage the mannose donor. An AlphaFold3-predicted acceptor-bound model further defines the structural basis of acceptor substrate recognition and suggests a plausible catalytic mechanism. Comparison with structural homologs highlights a distinct mode of substrate engagement by GPI-MT-I. Together, these findings establish a mechanistic framework for GPI-MT-I function with broader implications for the GPI-MT family.

## 1. Introduction

*Candida* infections pose a serious global health threat due to their high morbidity and mortality rates [1]. As opportunistic pathogens, *Candida* species primarily infect immunocompromised individuals, including patients with AIDS or cancer and those undergoing antibiotic or chemotherapy treatments [1]. Epidemiological studies estimate that invasive candidiasis and *Candida* bloodstream infections account for approximately 1.565 million cases annually worldwide, resulting in nearly 995,000 deaths (63.6%) [2]. Among these pathogens, *Candida glabrata* has emerged as one of the most clinically relevant species and is the second most common cause of candidemia in many regions worldwide [3]. Current first-line antifungal agents include polyenes, flucytosine, azoles, and echinocandins. However, the increasing prevalence of antifungal resistance complicates treatment and significantly limits therapeutic efficacy. In particular, the emergence of multidrug-resistant *C. glabrata* isolates exhibiting resistance to both azoles and echinocandins poses a severe challenge to current antifungal strategies [4,5]. Thus, beyond optimizing current antifungal regimens, the development of innovative therapeutic strategies is urgently required.

The GPI biosynthetic pathway is a crucial, conserved multi-step process in eukaryotes that produces GPI glycolipids. These glycolipids are essential for anchoring numerous proteins, known as GPI-anchored proteins (GPI-APs), to the cell surface [6,7]. In fungi, the biosynthesis of GPI is vital for maintaining the integrity of the cell wall and is closely linked to the pathogenicity and virulence of harmful fungi [8,9]. Disruption of this pathway impairs GPI anchor maturation, leading to proteostasis defects in the endoplasmic reticulum and potentially lethal cellular stress [10]. Several inhibitors targeting the GPI pathway have demonstrated significant antifungal activity [6,11]. Among these, inhibition of Gwt1 stands out due to its broad selectivity against fungal species while sparing the mammalian counterpart [12,13]. These findings highlight the GPI biosynthetic pathway as a promising target for the development of novel antifungal therapeutics.

The GPI anchor is structured around a core sequence: ethanolamine-PO4-(6)-Manα-(1-2)-Manα-(1-6)-Manα-(1-4)-GlcNα-(1-6)-myo-inositol-phospholipid. During synthesis, phosphatidylinositol (PI) undergoes several modifications involving glucosamine, an acyl group, mannoses (Man), and ethanolamine phosphates (EtNP) to form GPI. Following assembly of the GPI precursor, the terminal EtNP is covalently linked to the C-terminus of target proteins via an amide bond (Figure S1). The resulting GPI-APs undergo further processing in the ER and Golgi, including lipid remodeling and glycan modifications, before being trafficked to the plasma membrane and incorporated into the fungal cell wall [7,14,15]. In fungi, mannose addition is critical for GPI precursor assembly and for proper localization of GPI-APs. The three mannoses of the fungal GPI core are sequentially transferred by three mannosyltransferases: the Gpi14–Pbn1 complex (GPI-MT-I), the Gpi18–Pga1 complex (GPI-MT-II), and Gpi10 (GPI-MT-III) [16,17,18,19,20,21]. In addition, Smp3 introduces a fourth mannose, which is essential in yeast GPI biosynthesis [22]. Despite their importance, structural information on these GPI mannosyltransferases has remained lacking.

**Figure 1:**
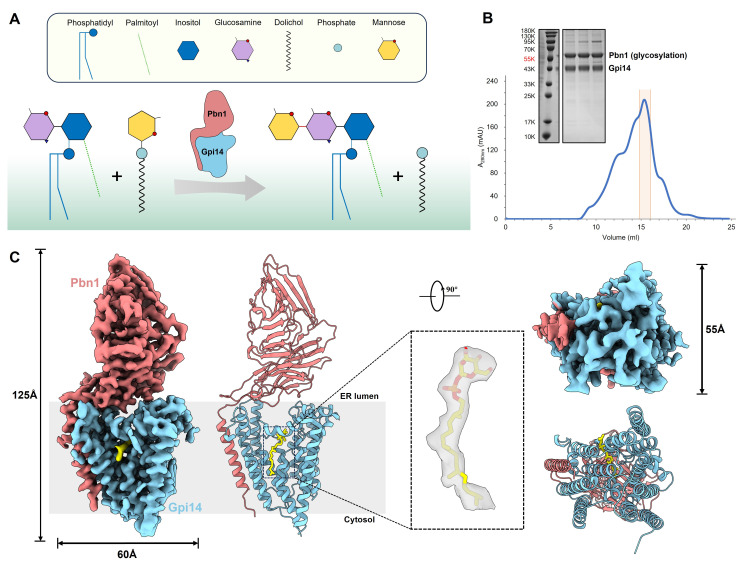
Cryo-EM structure of *Candida glabrata* GPI-MT-I in complex with Dol-P-Man. (**A**) Schematic illustration of the catalytic reaction, in which GPI-MT-I transfers the first mannose residue from Dol-P-Man to the GPI precursor. Key molecular components involved are highlighted in the inset. The pale green shading represents the endoplasmic reticulum (ER) membrane. (**B**) Size-exclusion chromatography profile and Coomassie blue-stained SDS-PAGE of purified GPI-MT-I complex. The complex consists of Gpi14 and glycosylated Pbn1. Fractions corresponding to the peak (orange box) were pooled for concentration. (**C**) Cryo-EM density map and cartoon model of GPI-MT-I, shown in side view (left) and bottom view (right). Gpi14 and Pbn1 are colored sky blue and light coral, respectively. The bound Dol-P-Man substrate is shown in yellow, with its cryo-EM density highlighted in the inset.

GPI-MT-I is a heterodimer composed of the catalytic subunit Gpi14 and the auxiliary subunit Pbn1, which together transfer the first mannose from dolichol-phosphomannose (Dol-P-Man) to GlcN-(acyl)PI, forming an α-1,4-glycosidic linkage [23] (Figure 1A). This enzyme complex is widely conserved among eukaryotes and plays a critical role in fungal growth, cell wall integrity, and hyphal morphogenesis [24,25]. Although genetic and biochemical studies have elucidated the functions of its subunits, the lack of structural information has hindered mechanistic understanding. Here, we report the cryo-EM structure of *Candida glabrata* GPI-MT-I bound to Dol-P-Man at 3.48 Å resolution, revealing how Gpi14 and Pbn1 assemble into a functional complex and engage the donor substrate. An AlphaFold3-predicted acceptor-bound model further defines the structural basis of acceptor recognition and suggests a plausible catalytic mechanism. Together, these findings uncover a distinct mode of substrate engagement by GPI-MT-I and establish a structural framework for understanding the glycosyltransferase family and guiding antifungal drug discovery.

## 2. Materials and Methods

### 2.1. Plasmid Construction

The full length *GPI14* gene (UniProt: Q6FXQ5) was amplified from the genome of *C. glabrata* ATCC 2001 and cloned into the p415GAL1 vector with a C-terminal 6×His tag. Similarly, the full length *PBN1* gene (UniProt: Q6FX62) was amplified and inserted into the p416GAL1 vector, incorporating an N-terminal tandem Twin-Strep II and FLAG tag.

### 2.2. Protein Expression and Purification

Gpi14 and Pbn1 were co-transformed into *S. cerevisiae* INVSc1 strain using the lithium acetate method [26]. Yeast cells were initially cultured in SC-Ura/Leu medium (Coolaber) supplemented with 2% glucose at 30 °C, with 200 rpm agitation for 24 h. Subsequently, they were added into YPG medium (containing 1% Yeast, 2% Peptone, and 2% D-Galactose) for an additional 24 h to induce protein over-expression. Yeast cells were harvested and suspended in a buffer consisting of 20  mM Hepes at pH 7.4, 150  mM NaCl with protease inhibitors, and then disrupted for six rounds by cell disruptor (AH-NANO, ATS, Suzhou, China). The cell debris was removed by low-speed centrifugation (4000× *g*) for 10 min. After centrifugation at 58,000× *g* for 1  h, the membranes were collected and solubilized by 1% lauryl maltose neopentyl glycol (LMNG) and 0.2% cholesterol hemisuccinate (CHS) for 1.5  h at 4 °C. Insoluble materials were removed by centrifugation at 58,000× *g* for 1  h and the detergent solubilized supernatant was then applied to Strep-Tactin resin (IBA). The resin was washed with buffer containing 20  mM Hepes at pH 7.4, 150  mM NaCl, 0.02% glyco-diosgenin (GDN) and eluted with buffer containing 20  mM Hepes at pH 7.4, 150  mM NaCl, 0.02% GDN, 2.5  mM desthiobiotin. The eluted protein was concentrated and subjected to further purification through size-exclusion chromatography (Superose 6 10/300 GL Increased, Cytiva, Marlborough, MA, USA). The peak fractions were analyzed by 15% SDS-PAGE. The purified sample was concentrated and stored at −80  °C for future use.

### 2.3. Cryo-EM Sample Preparation and Data Collection

3.5  µL of concentrated Gpi14-Pbn1 complex (~22  mg/mL) was applied to a glow-discharged holey carbon gold grid (R1.2/1.3, 400 mesh, Quantifoil, Großlöbichau, Germany), and then incubated for 30 s and subsequently blotted for 4 s at 8 °C and 100% humidity. The grids were subsequently plunge-frozen in liquid ethane cooled by liquid nitrogen using Thermo Fisher Vitrobot Mark IV (Waltham, MA, USA), and then loaded into an Thermo Fisher Titan Krios electron microscope operated at 300 kV. All movies were recorded using a K3 direct electron detector (Gatan, Pleasanton, CA, USA) at a nominal magnification of 81,000× (physical pixel size: 1.088 Å/pixel) using EPU software 2.10 for Gpi14-Pbn1 complex. The movies were collected with automated super resolution mode and dose-fractionated to 32 frames with a total exposure time of 3.2 s and total dose of 56 e^−^/Å^2^. Defocus ranges of −1.7 to −2.1 µm were used for all movies.

### 2.4. Cryo-EM Image Processing

For the Gpi14-Pbn1 complex dataset, beam-induced motion of 5008 movies were corrected by MotionCor2 1.5.0 [27]. Generated dosed-weighted micrographs were imported into cryoSPARC v4.0.3 [28] to determine CTF parameters through patch-CTF. Hundreds of micrographs were pre-processed to generate good and bad initial 3D references and a total of 43,298 particles were picked to train a Topaz model. After Topaz extract, 1,141,564 particles were obtained for further 2D classification. After removing undesirable particles through iterative rounds of heterogeneous refinement with C1 symmetry, 80,856 particles were retained. After applying non-uniform refinement, we eventually obtained the cryo-EM maps of GPI-MT-I at resolutions of 3.48 Å, based on the ‘gold standard’ Fourier shell correlation (FSC) = 0.143. Local-resolution estimations were performed by cryoSPARC.

### 2.5. Model Building

The Gpi14 and Pbn1 models predicted by AlphaFold3 [29], along with the Dol-P-Man molecule, were fitted into the cryo-EM map using ChimeraX 1.5 [30] and manually adjusted in Coot 0.9.8 [31] based on the density. The resulting model then underwent real space refinement in Phenix 1.20.1 [32]. The restraints file of Dol-P-Man was generated by eLBOW 1.20.1-4487 [33] in Phenix. The geometries of all models were evaluated by MolProbity (http://molprobity.biochem.duke.edu/) [34]. Cryo-EM data collection, refinement and validation statistics are presented in Table S1.

## 3. Results

### 3.1. Structural Determination of GPI-MT-I Complex from C. glabrata

The full-length *C. glabrata* Gpi14 (431 amino acids) and Pbn1 (416 amino acids) were co-expressed recombinantly in *Saccharomyces cerevisiae*. Initially, the membrane protein complex was solubilized using LMNG-CHS detergent, and subsequently, it was exchanged into GDN via a strep-affinity column facilitated by Pbn1. The peak fractions obtained from size-exclusion chromatography exhibited high purity, containing both subunits Gpi14 and Pbn1, as observed through Coomassie staining (Figure 1B). Similar stable Gpi14-Pbn1 complexes have been observed in other eukaryotes, including humans and protists such as *Trypanosoma brucei* and *Leishmania* [35,36,37], indicating that the assembly of this heterodimeric complex is conserved across diverse species.

To elucidate the assembly and mechanism of the GPI-MT-I complex, we conducted single-particle cryo-EM analysis on the highly purified Gpi14-Pbn1 complex. Through standard sample preparation and data processing protocols, we achieved reconstructed cryo-EM map of the Gpi14-Pbn1 complex at an overall resolution of 3.48 Å (Figure S2). This map exhibited high-quality densities, facilitating the generation of de novo models for both Gpi14 and Pbn1 (Figure S3). Our final model comprised residues 2-408 of Gpi14 and residues 2-408 of Pbn1. The overall structure of Gpi14-Pbn1 complex measures approximately 60  Å  ×  55  Å  ×  125  Å and reveals a 1:1 heterodimer of Gpi14 and Pbn1 (Figure 1C). Within Pbn1, we identified three N-glycosylation sites (N35, N319, and N365) (Figure S3C), consistent with observed band upshift in SDS-PAGE analysis. Additionally, a prominent glycolipid-like density was observed, indicative of a Dol-P-Man molecule (Figure 1C). By modeling Dol20-P-Man into this density, we successfully obtained the high-resolution structure of the endogenous donor-bound GPI-MT-I complex.

### 3.2. Architecture of GPI-MT-I Complex

**Figure 2:**
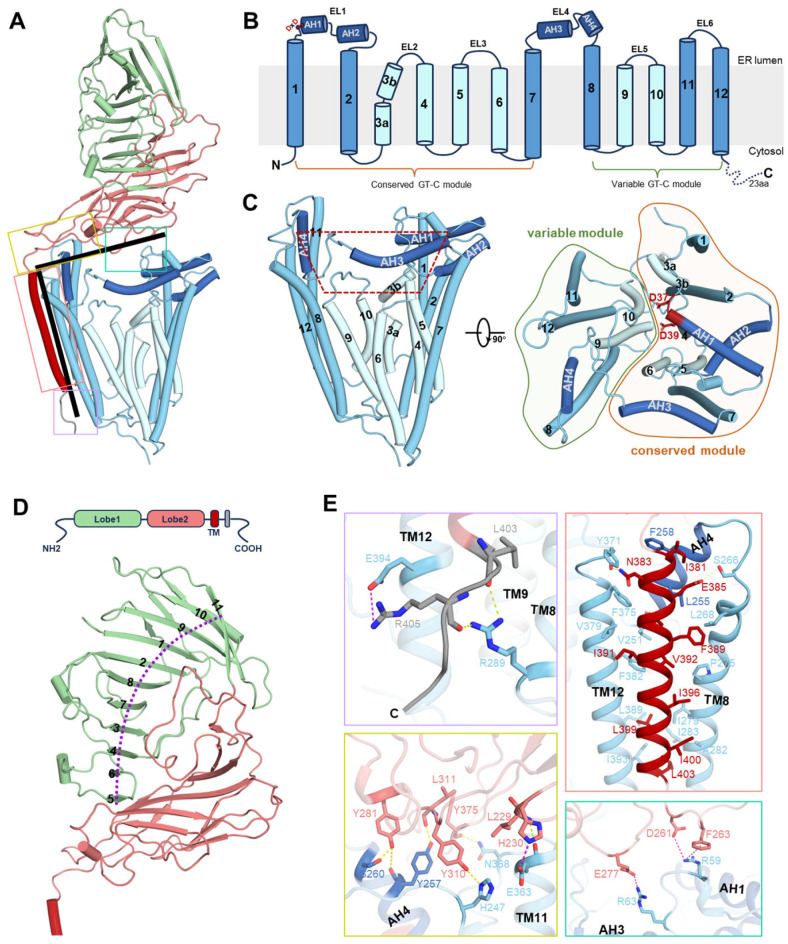
Architecture of the Gpi14-Pbn1 complex. (**A**) Overall structure of the Gpi14-Pbn1 complex, viewed parallel to the membrane. The L-shaped interfaces between subunits are boxed. (**B**) Topological diagram of Gpi14. The conserved DxD motif critical for catalysis is marked with a red dot. (**C**) Side view (left) and top view (right) of Gpi14. Helices are shown as cylinders, colored as in (**B**). The membrane core region (cyan) is formed by short transmembrane helices (TMHs), surrounded by a ring of long TMHs (sky blue), creating a trapezoidal transmembrane cavity (outlined by a red dashed line). The conserved GT-C module is shaded in orange, and the variable module in green. (**D**) Schematic representation of Pbn1. Its luminal domain (Lobe 1 and Lobe 2), single TMH, and cytoplasmic tail are shown in distinct colors. Lobe 1 contains a central “rib-cage” β-sheet (purple curve, β1–β11). (**E**) Close-up views of Gpi14-Pbn1 interactions corresponding to the boxed regions in (**A**). Contacts are grouped into cytoplasmic, transmembrane, and luminal interactions. Lobe 2 establishes two distinct interfaces with Gpi14. Interacting residues are shown as sticks.

Gpi14 has 12 transmembrane helices, with both the N-terminus and the C-terminus located in the cytoplasm (Figure 2A–C). Two long external loops (EL1 and EL4) face the endoplasmic reticulum (ER) lumen and contain four amphipathic helices (AH1-4) (Figure 2B,C). Gpi14 features a membrane core region with short transmembrane helices (TMHs) enclosed by a ring of long TMHs (Figure 2C). This arrangement creates a trapezoidal membrane void that accommodates the four amphipathic helices and contributes to the formation of the substrate catalytic center (Figure 2C). According to previous classification [38], Gpi14 is a glycosyltransferase of the C-superfamily (GT-C), characterized by a typical modular architecture. Its N-terminal region (TM1-7) constitutes the conserved module, whereas the C-terminal region (TM8-12) forms the variable module (Figure 2C). Notably, Gpi14 contains a canonical DxD motif oriented toward the ER lumen, which is conserved across species (Figure 2B,C and Figure S4). Previous mutational analyses have demonstrated that both aspartate residues within this motif are indispensable for GPI-MT-I catalytic activity, with substitution of D51 in human PIG-M (corresponding to D39 in the *C. glabrata* ortholog) exerting a more pronounced effect [17].

Pbn1 is an endoplasmic reticulum (ER)-resident type I membrane glycoprotein with a single C-terminal transmembrane helix (Figure 2D). This helix anchors Pbn1 to the outer surface of Gpi14, positioning its C-terminus toward the cytoplasm, while the N-terminus extends into the ER lumen to form a large soluble domain comprising two structurally distinct lobes (Figure 2A,D). Lobe 1 features a core of eleven twisted antiparallel β-strands arranged into a “half-rib” cage, with its exterior decorated by loops, short α-helices, and a three-stranded β-sheet (Figure 2D). Interestingly, this lobe is absent in the homologs PIGX (human) and Pbn1 (*T. brucei*), where it is replaced by a canonical N-terminal signal peptide that targets the proteins to the ER membrane (Figure S5A,B). This structural difference suggests that yeast Pbn1 follows a non-classical ER insertion pathway, likely post-translational, consistent with its C-terminal membrane anchor [39]. By contrast, lobe 2 is composed of two β-sheets and functions as the principal Gpi14-interaction domain (Figure 2A,D). It also forms a hydrophobic groove that encloses lobe 1, whereas in other species the corresponding interface is predominantly hydrophilic (Figure S5C).

The interaction between Gpi14 and Pbn1 is mediated through multiple interfaces, adopting an L-shaped configuration with a total buried surface area of more than 2300 Å^2^ (Figure 2A). On the cytosolic side, electrostatic and hydrogen-bond interactions mediate contacts between defined regions of Pbn1 and Gpi14 (Figure 2E). Within the membrane, the single transmembrane helix of Pbn1 packs extensively against TM8 and TM12 of Gpi14, as well as AH4 of EL4, through hydrophobic interactions that are further reinforced by polar contacts (Figure 2E). On the luminal side, lobe 2 engages directly with Gpi14, while lobe 1 remains largely peripheral (Figure 2A). Two distinct interfaces contribute to this luminal interaction: (i) a broad membrane-proximal interface at the corner of the L-shaped structure, where multiple loops of Pbn1 dock onto Gpi14 to create a stable contact surface, and (ii) a smaller, localized interface characterized by point-to-point electrostatic and π-cation interactions (Figure 2E). Together, these multivalent interactions secure the stable assembly to form functional complex within the ER membrane.

### 3.3. Donor Recognition by GPI-MT-I Complex

**Figure 3:**
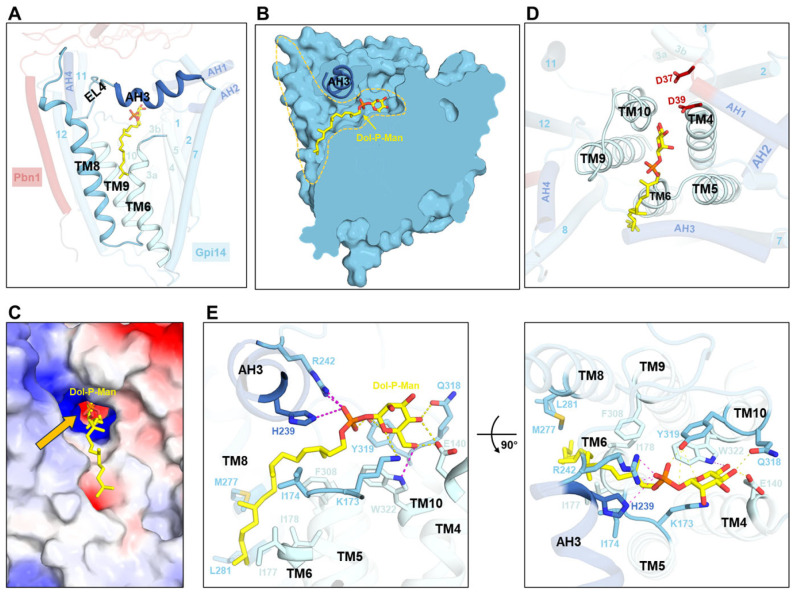
Donor recognition by GPI-MT-I. (**A**) Donor-binding site of GPI-MT-I. Gpi14 and Pbn1 are shown as cylinders, with the TMHs forming the donor portal depicted as cartoons. Dol-P-Man is shown as yellow sticks. (**B**) A funnel-shaped channel within Gpi14 guides donor entry. Gpi14 is shown in a clipped view, with AH3 highlighted in cartoon representation. (**C**) Electrostatic surface potential of GPI-MT-I, colored red (negative), white (neutral), and blue (positive). Bound Dol-P-Man is shown as sticks, and orange arrows highlight the proximity of its phosphate groups to positively charged surfaces. (**D**) Top view of donor-bound GPI-MT-I. The mannose moiety of Dol-P-Man is positioned at the geometric center of the inner core formed by TM4, TM5, TM6, TM9, and TM10. (**E**) Coordination of Dol-P-Man within the Gpi14 transmembrane region. The donor substrate and interacting residues are shown as sticks, with electrostatic and hydrogen-bonding interactions indicated by magenta and yellow dashed lines, respectively.

The donor substrate Dol20-P-Man is embedded within the transmembrane cavity of Gpi14, oriented toward the EL4 side, and does not directly interact with Pbn1 (Figure 1C and Figure 3A). For donor substrate entry, the upper portions of TM6, TM8, and TM9, together with EL4 segment, form an open door-like architecture that guides Dol20-P-Man into the funnel-shaped channel (Figure 3A,B). On the ER-luminal side, the horizontal AH3 helix and its adjacent loop region create an arch above the groove (Figure 3A,B). This structural constraint induces the dolichol moiety bend at the membrane boundary, ensuring that the phosphate group remains close to the positively charged surface (Figure 3B,C). The dolichol moiety is attached to a hydrophobic groove on the surface of Gpi14, close to the upper region of TM6 (Figure 3C,D). The mannose moiety is positioned at the geometric center of the trapezoidal membrane void, suspended above the inner core formed by short transmembrane helices (Figure 3D).

Recognition of Dol-P-Man substrates is mediated through a combination of hydrophobic, electrostatic, and hydrogen-bonding interactions (Figure 3E). The dolichyl tail forms hydrophobic interactions with Ile174 of EL3, Ile177 and Ile178 of TM6, Met277 and Leu281 of TM8, and Phe308 of TM9 (Figure 3E). The phosphate group forms electrostatic interactions with the side chains of His239 and Arg242, as well as hydrogen bonds with the phenolic hydroxyl group of Tyr319 (Figure 3E). Meanwhile, the mannosyl group is coordinated through an extensive hydrogen-bonding network (Figure 3E). The ring oxygen forms hydrogen bonds with the side chains of Lys173 and Tyr319 (Figure 3E). The C4-linked hydroxyl group forms hydrogen bonds with Glu140 and Gln318, while the C6 hydroxyl group engages in both a hydrogen bond with Glu140 and an electrostatic interaction with Trp322 (Figure 3E). This multi-faceted recognition mechanism ensures precise substrate positioning for catalysis. Notably, most of these residues exhibit a high degree of evolutionary conservation, highlighting their essential contributions to dolichol binding affinity and proper localization within the catalytic pocket (Figure S4).

### 3.4. AlphaFold3-Predicted Structure of the Acceptor-Bound GPI-MT-I Complex

To elucidate the mechanism by which GPI-MT-I recognizes its acceptor substrate, AlphaFold3 [29] was employed to predict the structure of the enzyme-acceptor complex. As a validation step, the structures of GPI-MT-I alone and in the donor-bound state were first predicted and compared with the experimentally determined cryo-EM structure (Figure S6). The high structural similarity confirmed the reliability of the prediction (Figure S6B,C). Notably, the Dol-P-Man-bound state observed in our cryo-EM map is positioned slightly closer to the catalytic center than in the predicted model (Figure S6C). Building on this validation, the ternary complex of GPI-MT-I bound to its acceptor substrate GlcN-(acyl)PI was then predicted, yielding a high-confidence structural model (Figure S6A).

**Figure 4:**
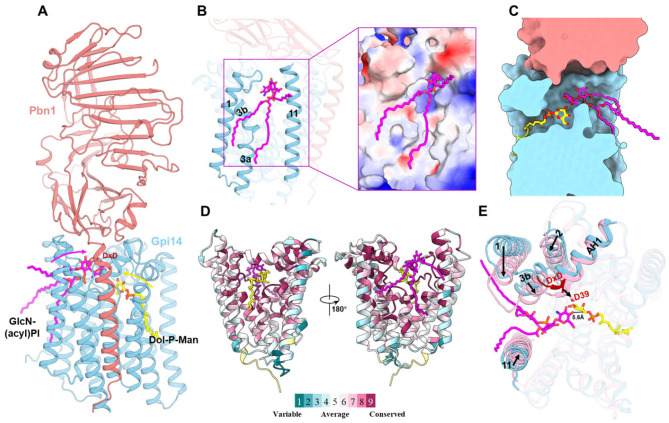
AlphaFold3-predicted acceptor substrate binding by GPI-MT-I. (**A**) Binding of donor and acceptor substrates within Gpi14. The donor Dol-P-Man (yellow) enters the catalytic center from the side opposite to the acceptor GlcN-(acyl)PI (magenta). Colored arrows indicate the respective entry pathways, with the acceptor positioned above the donor. (**B**) The acceptor entry site. Cartoon view (left) and electrostatic surface potential (right) highlight the entrance, with the hydrophilic head of the acceptor inserted into the complex and its hydrophobic tail aligned along the hydrophobic surface of Gpi14. (**C**) Sectional view of the continuous catalytic channel within GPI-MT-I. Gpi14 and Pbn1 are shown as surfaces, while the donor and acceptor are shown as sticks. (**D**) Sequence conservation of Gpi14 calculated by ConSurf [43]. Conserved regions are colored maroon, variable regions cyan. (**E**) Top view of Gpi14 bound to different substrates. Acceptor binding induces rearrangements of TM1, TM2, TM3b, and TM11, together with a pronounced conformational shift in the DxD motif, particularly residue D39.

The acceptor substrate GlcN-(acyl)PI, similar to the donor Dol20-P-Man, binds to Gpi14 but occupies the site opposite to the donor-binding pocket (Figure 4A). The acceptor entry site of GPI-MT-I is primarily defined by TM1, TM3b, and TM11 (Figure 4B). At this site, the hydrophilic headgroup of GlcN-(acyl)PI penetrates into the interior of the complex, whereas its hydrophobic tail aligns along a surface groove of Gpi14 (Figure 4B). Together, Gpi14 and Pbn1 establish a continuous catalytic channel that accommodates both donor and acceptor substrates on the luminal side of the ER (Figure 4C). The substrate-recognition regions of Gpi14 are highly conserved (Figure 4D), supporting the notion that the catalytic mechanism of Gpi14-mediated mannosyl transfer is evolutionarily preserved.

Structural comparisons reveal distinct conformational differences in Gpi14 between the donor-bound (Dol-P-Man) and acceptor-bound (GlcN-(acyl)PI) states (Figure 4E). Binding of the acceptor substrate induces pronounced rearrangements at the acceptor-binding site, most notably shifts in TM1-3 and TM11 toward the substrate (Figure 4E). Concomitantly, the catalytic DxD motif repositions toward the GlcN moiety (Figure 4E). In particular, residue D39 moves into proximity with the C4 hydroxyl group of GlcN (Figure 4E), where it functions as the catalytic base to deprotonate this hydroxyl, generating a C4 alkoxide. This activated C4 alkoxide then serves as a nucleophile to attack the electrophilic C1 phosphate of Dol-P-Man, facilitating formation of the α-1,4 glycosidic bond (Figure S7). However, in the current structure, the distance between the GlcN C4 alkoxide and the donor C1-phosphate bond remains relatively long (~5.6 Å) (Figure 4E), suggesting that further conformational adjustments of the Dol-P-Man headgroup are required to achieve an optimal geometry for catalysis. Similar acceptor-induced conformational transitions have been reported in other GT-C family enzymes, including DYP19, PglB and Alg6 [40,41,42].

### 3.5. Comparative Analysis of GPI-MT-I Complex with Its Structural Homologs

**Figure 5:**
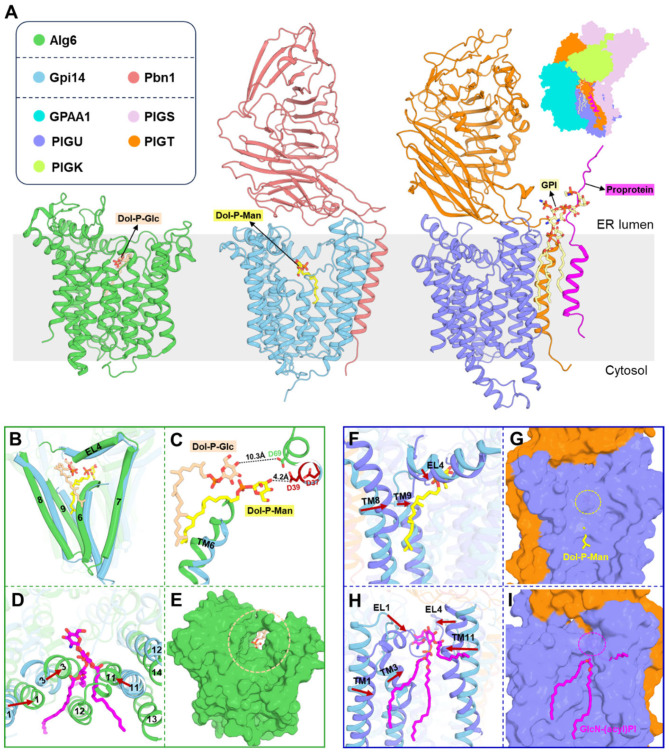
Structural comparison of GPI-MT-I with its homologs. (**A**) Overall structures of GPI-MT-I, Alg6, and the PIGU-PIGT complex. Protein subunits are shown as cartoons in distinct colors, ligands as sticks. The ER membrane is shaded grey. (**B**) Donor recognition by Gpi14 (sky blue) versus Alg6 (green). Transmembrane helices are shown as cylinders. Dol-P-Man and Dol-P-Glc are depicted as yellow and wheat sticks, respectively. (**C**) Close-up of the donor-binding sites shown in (**B**). In Alg6, the Dol-P-Glc hydrophobic tail adopts a distinct turn, while its hydrophilic moiety is positioned further from D69, the catalytic base, compared with GPI-MT-I. (**D**) Top view of GPI-MT-I acceptor binding mapped onto Alg6. In Alg6, TM1, TM3, TM11, and TM12 shift substantially and pack tightly, occluding the acceptor entry site of Gpi14. (**E**) Side view of Alg6. The wheat dashed circle marks the open acceptor-binding site and catalytic center. (**F**) Structural superposition of PIGU-PIGT and Gpi14-Pbn1 complexes from the donor side. TM8, TM9, and EL4 of PIGU-PIGT shift closer to Dol-P-Man. (**G**) Surface view of PIGU-PIGT from the donor side. The Dol-P-Man entrance (yellow dashed circle) is fully closed. (**H**) Structural superposition of PIGU-PIGT and Gpi14-Pbn1 complexes from the acceptor side. TM1, TM3, TM11, EL1, and EL4 of PIGU-PIGT are displaced toward GlcN-(acyl)PI. (**I**) Surface view of PIGU-PIGT from the acceptor side. The GlcN-(acyl)PI entrance (magenta dashed circle) is completely blocked.

A Dali search [44] of the Protein Data Bank (PDB) identified Alg6 [42], a glucosyltransferase, as a structural homolog of Gpi14, and revealed that the Gpi14-Pbn1 complex adopts a three-dimensional architecture resembling the PIGU-PIGT subcomplex of the GPI transamidase (GPI-T) complex [45] (Figure 5A). Despite these structural similarities, Gpi14-Pbn1, Alg6, and PIGU-PIGT differ markedly in biological function, assembly state, and substrate recognition. All three are localized to the ER membrane; however, Gpi14-Pbn1 and PIGU-PIGT contribute to GPI-anchored protein biosynthesis, whereas Alg6 participates in protein N-glycosylation [17,42,45]. Alg6 functions as a monomeric enzyme, whereas GPI-MT-I operates as a heterodimer comprising the catalytic subunit Gpi14 and the auxiliary subunit Pbn1, which act in concert to achieve full enzymatic activity [46]. By contrast, GPI-T is a pentameric complex, in which PIGU and PIGT form a scaffold that supports the remaining subunits, including the catalytic component PIGK [45,47] (Figure 5A).

Comparative structural analysis revealed distinct features of glycosyl donor recognition in GPI-MT-I and Alg6, consistent with their divergent substrate specificities. Both enzymes utilize dolichol-phosphate-linked sugar donors, yet their reactions differ: Alg6 transfers glucose from Dol-P-Glc to form an α-1,3-glycosidic linkage, whereas Gpi14 mediates mannose transfer from Dol-P-Man to generate an α-1,4-glycosidic bond [23,42]. Despite their broadly similar transmembrane helix arrangements and donor portal architectures, Dol-P-Man binds more deeply within Gpi14 (Figure 5A,B). As a result, the mannose moiety in Gpi14 is positioned significantly closer to the catalytic site (4.2 Å from Asp39) than glucose in Alg6 (10.3 Å from Asp69) (Figure 5C). Acceptor recognition further distinguishes the two enzymes. Alg6 contains 14 transmembrane helices, with TM1, TM3, TM11, and TM12 arranged compactly to occlude the substrate entry site present in Gpi14 (Figure 5D). In particular, TM11 and TM12 introduce substantial steric hindrance that likely prevents accommodation of the GPI-MT-I acceptor substrate (Figure 5D). Furthermore, Alg6 lacks a substantial ER luminal domain, leading to near-complete exposure of its catalytic center and providing greater conformational flexibility for acceptor engagement (Figure 5E).

Although PIGU possesses the same number of transmembrane helices as Gpi14, their more compact arrangement gives rise to a closed surface devoid of substrate channels (Figure 5F–I), indicating a different mode of substrate recognition. In PIGU, TM8, TM9, and EL4 undergo substantial conformational rearrangements that obstruct the donor entry site (Figure 5F,G). In parallel, TM1 and TM3 exhibit slight positional shifts, whereas TM11 and the loops of EL1 and EL4 undergo more pronounced conformational changes, effectively blocking the acceptor portal corresponding to that of GPI-MT-I (Figure 5H,I). Moreover, the catalytic channel observed in Gpi14-Pbn1 is completely occluded in the PIGU-PIGT complex, and the catalytically essential DxD motif in EL1 is substituted by an SSW sequence, suggesting a fundamentally different mechanism of action (Figure S8). Structural analysis confirms that both substrates of GPI-T bind on the side of the transmembrane helical bundle formed by PIGU and PIGT (Figure 5A). Collectively, these findings indicate that GPI-MT-I may employ a markedly distinct substrate recognition and catalytic mechanism from that of its structural homologs.

## 4. Discussion

The Gpi14-Pbn1 complex catalyzes the transfer of the first α-1,4-linked mannose to GlcN-(acyl)PI during GPI anchor precursor assembly [23], a process essential for fungal viability and thus representing a critical antifungal drug target. Understanding the assembly, substrate recognition, and catalytic mechanism of the Gpi14-Pbn1 complex is fundamental for the development of targeted therapeutics. In this study, we report the first high-resolution cryo-EM structure of the GPI-MT-I complex from *Candida glabrata*, a major human fungal pathogen, in complex with endogenous donor substrate Dol-P-Man. Integrating our experimental structure with the AlphaFold3-predicted acceptor-bound GPI-MT-I complex, we reveal the molecular assembly of the Gpi14-Pbn1 complex and elucidate the structural basis for its specific recognition of glycosyl donor and acceptor substrates, as well as the potential catalytic mechanism. Compared with structural homologs, the GPI-MT-I complex exhibits distinct substrate recognition mode. Collectively, these findings provide a detailed understanding of the GPI-MT-I complex and offer insights applicable to other GPI-MT family members.

**Figure 6:**
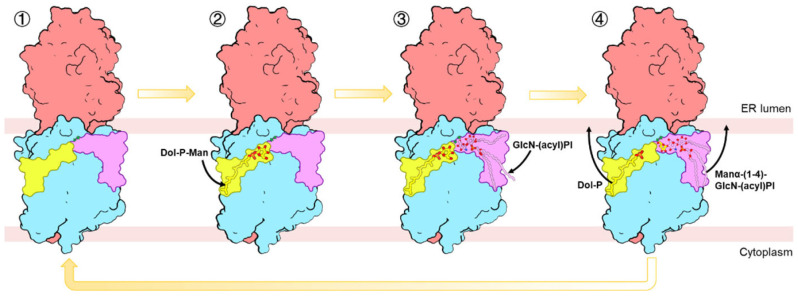
Proposed model of substrate recognition and catalysis by the GPI-MT-I. In the resting state, the donor substrate Dol-P-Man (yellow sticks) enters from one side of the complex. The acceptor substrate GlcN-(acyl)PI (magenta sticks), generated in the preceding step of GPI biosynthesis, enters from the opposite side. Acceptor binding triggers conformational rearrangements in Gpi14, repositioning D39 to function as the catalytic base and simultaneously inducing local adjustments of Dol-P-Man for catalysis. Following mannose transfer, Dol-P is released together with Manα-(1-4)-GlcN-(acyl)PI, completing one catalytic cycle.

Based on our cryo-EM structure in combination with AlphaFold3 predictions, we propose a mechanistic model for substrate recognition and catalysis by the Gpi14-Pbn1 complex (Figure 6). In this model, the donor substrate Dol-P-Man initially enters the GPI-MT-I complex from one side. Subsequently, the acceptor substrate GlcN-(acyl)PI, generated in the preceding step of GPI biosynthesis, enters from the opposite side. Its binding triggers a conformational rearrangement of Gpi14 that repositions D39 into hydrogen-bonding proximity of the C4 hydroxyl group, thereby facilitating its deprotonation and formation of a reactive C4 alkoxide. Concurrently, Dol-P-Man undergoes a local adjustment that positions its C1 phosphate for nucleophilic attack. The activated C4 alkoxide then attacks the electrophilic C1 phosphate of Dol-P-Man, driving formation of the α-1,4 glycosidic bond. Following catalysis, Dol-P is released together with the mannose-modified GlcN-(acyl)PI, completing a single cycle of mannose transfer.

Obtaining the acceptor substrate GlcN-(acyl)PI and its mannose-linked product remains a major challenge. Consequently, donor-acceptor-bound Gpi14-Pbn1 structure and product-bound states cannot yet be experimentally resolved. The unavailability of GlcN-(acyl)PI also prevents the establishment of robust in vitro biochemical systems to directly validate the catalytic mechanism. Collaborative efforts with synthetic chemists to produce these complex glycolipid molecules will be critical for achieving a comprehensive mechanistic understanding in the future. Nonetheless, our study substantially advances the current understanding of GPI mannosyltransferases and provides a strong foundation for the development of antifungal agents targeting GPI-MT-I complex.

## Data Availability

The density map of Gpi14-Pbn1 has been deposited in the EMDB under accession code EMD-66120, and the corresponding atomic model has been deposited in the Protein Data Bank under accession code 9WNO.

## Supplementary Materials

The following supporting information can be downloaded at: https://www.mdpi.com/article/10.3390/jof11110819/s1, Figure S1: Overview of GPI-anchored protein (GPI-AP) biosynthesis in yeast; Figure S2: Cryo-EM image processing of GPI-MT-I; Figure S3: Representative cryo-EM densities of GPI-MT-I; Figure S4: Multiple sequence alignment of Gpi14; Figure S5: Structural comparison of yeast Pbn1 with its homologs from human and *Trypanosoma brucei*; Figure S6: GPI-MT-I structures in different states predicted by AlphaFold3; Figure S7: Proposed catalytic mechanism of GPI-MT-I; Figure S8: Catalytic channel occlusion in the PIGU–PIGT complex; Table S1: Cryo-EM data collection, refinement, and validation statistics.
