# Supplementary material for: Structural Insights into the Glycosylphosphatidylinositol Mannosyltransferase I Complex from Candida glabrata
Source: J Fungi (Basel). 2025 Nov 19;11(11):819. doi: 10.3390/jof11110819 (PMC12653191; doi:10.3390/jof11110819)
Supplement: Supplementary file 1 [file jof-11-00819-s001.zip › jof-3957986-supplementary.pdf]

Structural Insights into the Glycosylphosphatidylinositol Mannosyltransferase I Complex from Candida glabrata

Hui Sun [1,†], Weihong Wu [1,†], Xiaomei Li [2,†], Yang Deng [3], Jiarong Huang [1], Meng Yin [1,*] and Zhaofeng Yan [1,*]

[1] School of Biomedical Sciences, Hunan University, Changsha 410082, China
[2] Shanxi Academy of Advanced Research and Innovation, Taiyuan 030032, China
[3] Hunan Provincial Key Laboratory of Anti-Resistance Microbial Drugs, The Third Hospital of Changsha, Changsha 410118, China
* Correspondence: yinm@hnu.edu.cn (M.Y.); zhaofengyan@hnu.edu.cn (Z.Y.)
† These authors contributed equally to this work.

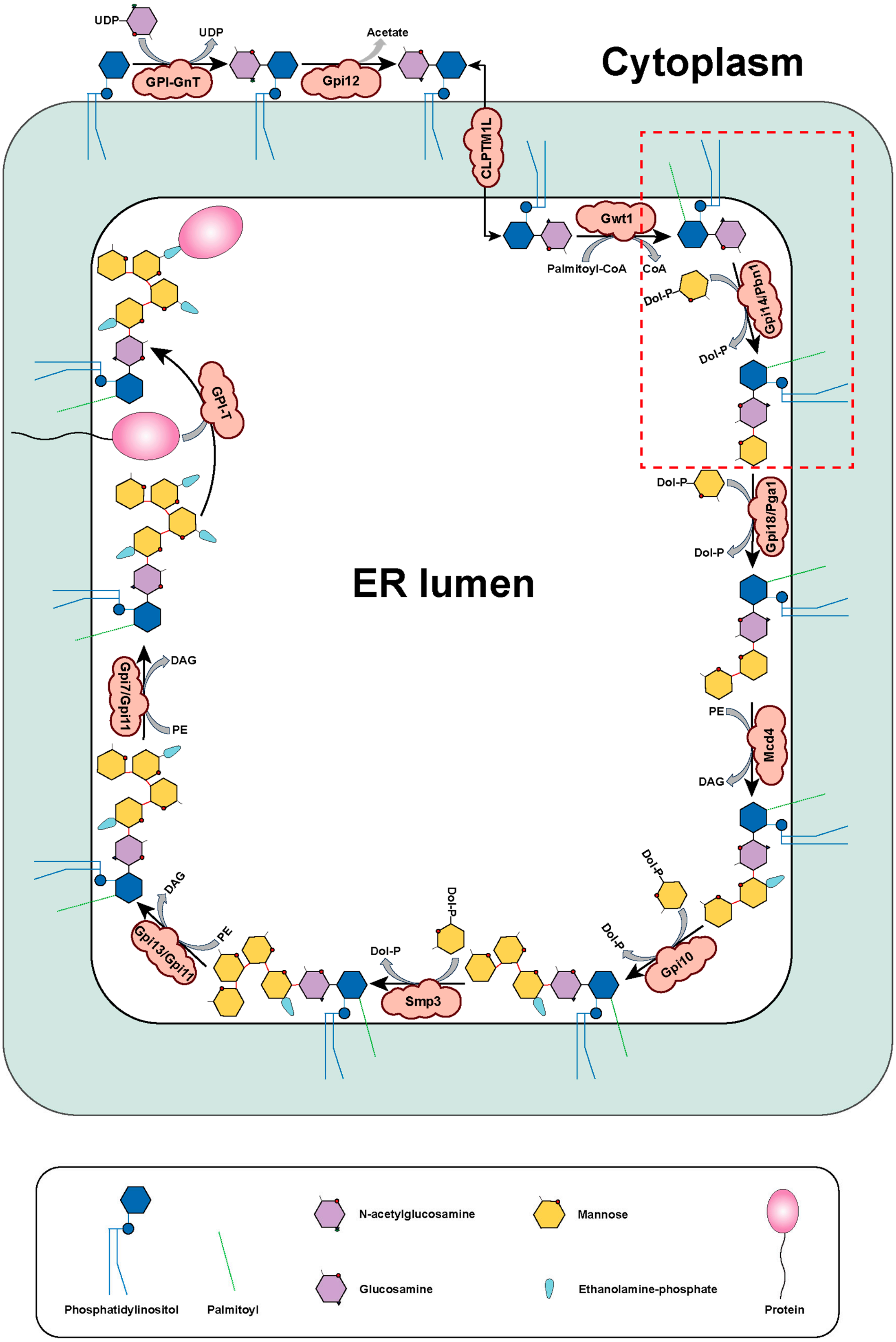

Figure S1. Overview of GPI-anchored protein (GPI-AP) biosynthesis in yeast. GPI biosynthesis is initiated on the cytoplasmic side of the endoplasmic reticulum (ER) and completed in the ER lumen. First, N-acetylglucosamine (GlcNAc) is transferred

from UDP-GlcNAc to phosphatidylinositol (PI) by the GPI-GlcNAc transferase (GPI-GnT) complex, yielding GlcNAc-PI. This intermediate is deacetylated by Gpi12 to form GlcN-PI, which is subsequently flipped into the ER lumen by CLPTM1L. Within the lumen, a series of modifications occur: inositol acylation by Gwt1; mannose additions by Gpi14/Pbn1 (mannosyltransferase I), Gpi18/Pga1 (mannosyltransferase II), Gpi10 (mannosyltransferase III), and Smp3 (mannosyltransferase IV); and phosphoethanolamine (EtNP) transfers by Mcd4 (EtNP transferase I), Gpi13/Gpi11 (EtNP transferase III), and Gpi7/Gpi11 (EtNP transferase II). These steps use dolichol-phosphomannose (Dol-P-Man) and phosphatidylethanolamine (PE) as donors. Finally, the mature GPI precursor is transferred to proteins bearing a GPI signal sequence by the GPI transamidase (GPI-T) complex, composed of Gpi8, Gaa1, Gpi17, Gpi16, and Gab1, in which Gpi8 functions as the catalytic subunit. GPI-T covalently links the preassembled GPI to the target protein via the amino group of the phosphoethanolamine attached to the third mannose.

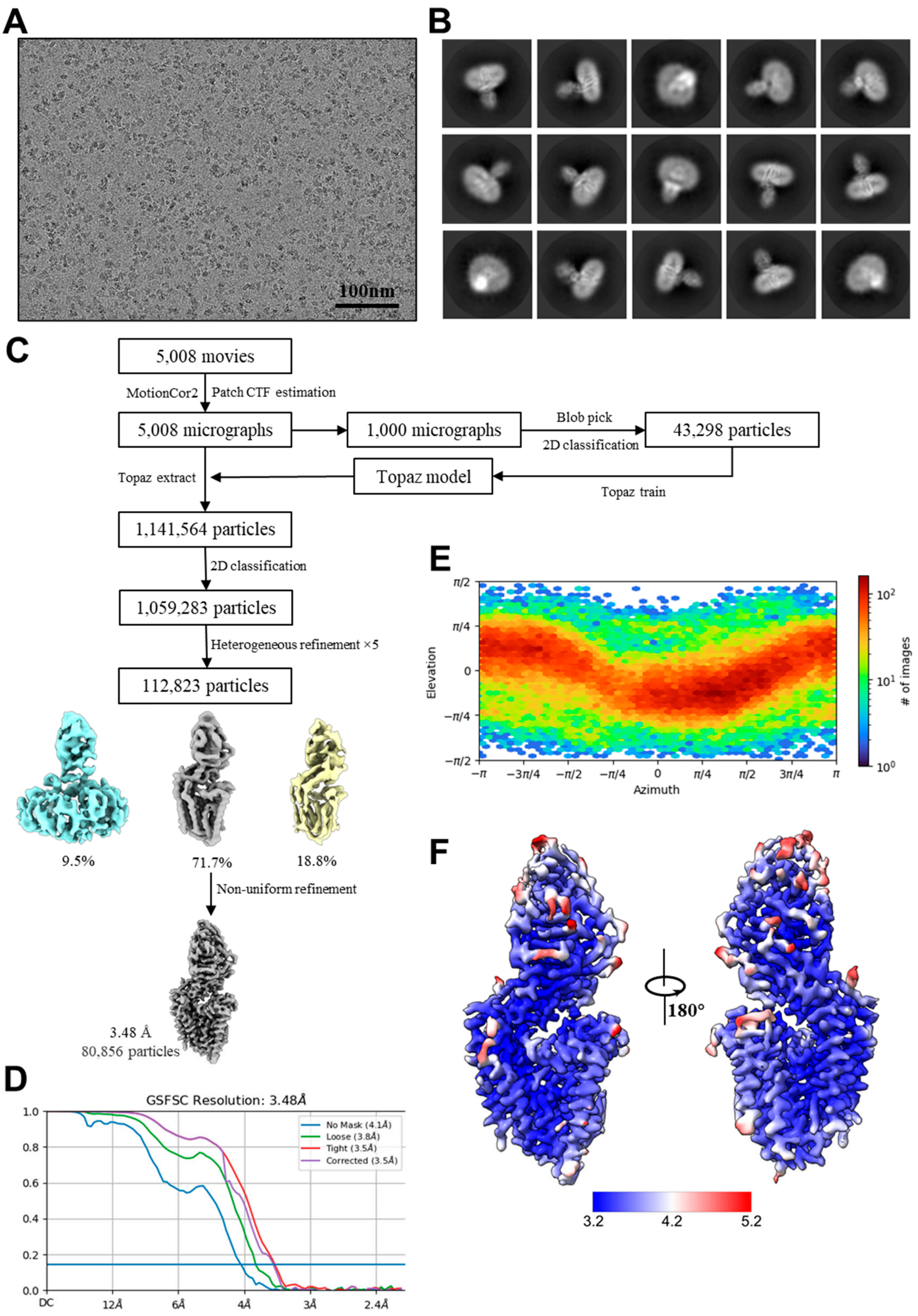

Figure S2. Cryo-EM image processing of GPI-MT-I.

(A) Representative cryo-EM micrograph of GPI-MT-I. Scale bar, 100 nm. **(B)** Representative 2D class averages of GPI-MT-I particles. **(C)** Image processing workflow of the GPI-MT-I dataset, yielding a focused refinement at 3.48 Å resolution.

(D) Gold-standard FSC curves of maps without mask (blue), with loose mask (green), tight mask (red), and corrected mask (purple). **(E)** Angular distribution of particles included in the final reconstruction. **(F)** Local resolution estimation of the GPI-MT-I density map.

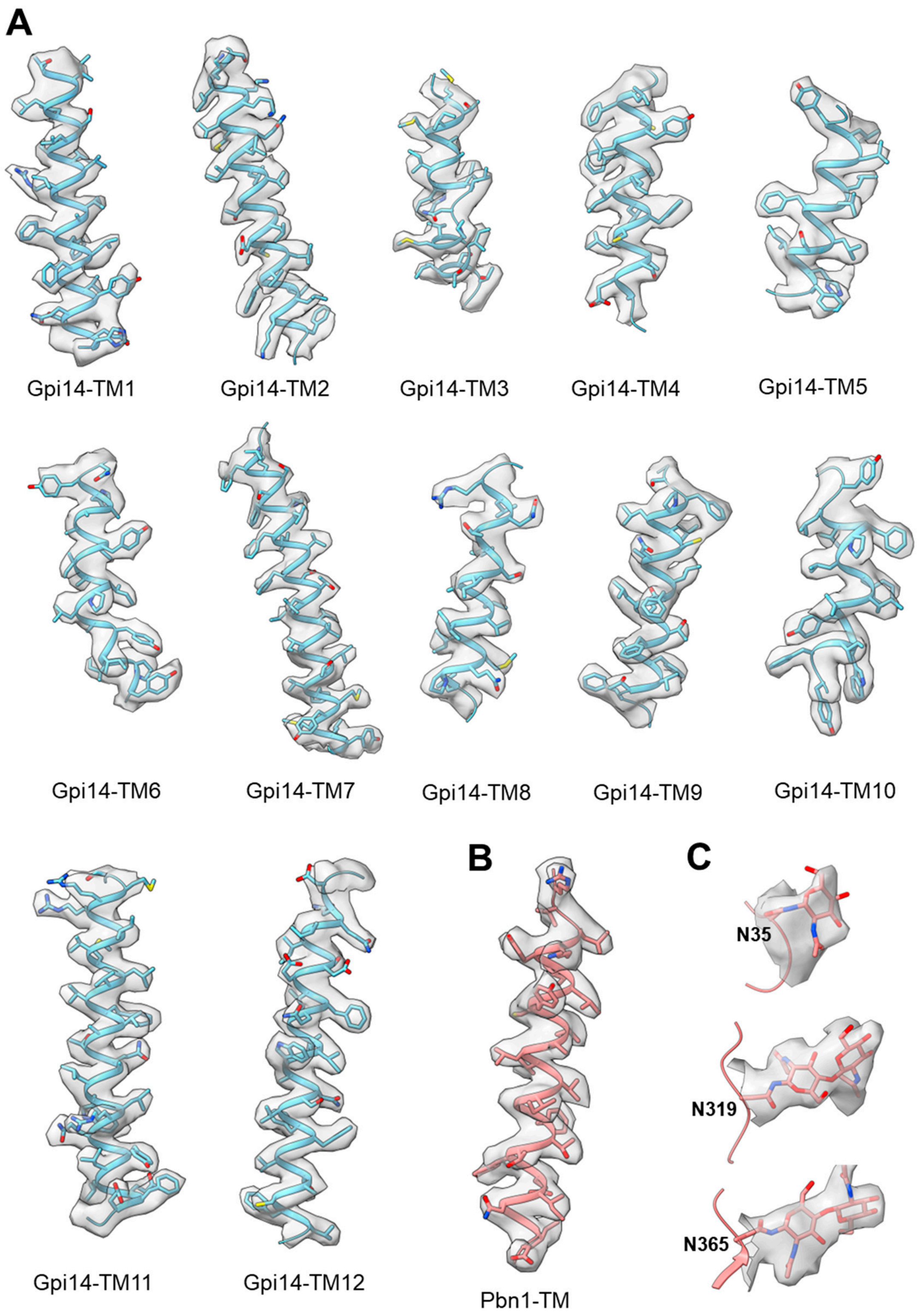

Figure S3. Representative cryo-EM densities of GPI-MT-I.

(A) Densities of the transmembrane helices of Gpi14. **(B)** Density of the single TM helix of Pbn1. **(C)** Densities of N-glycans associated with Pbn1.

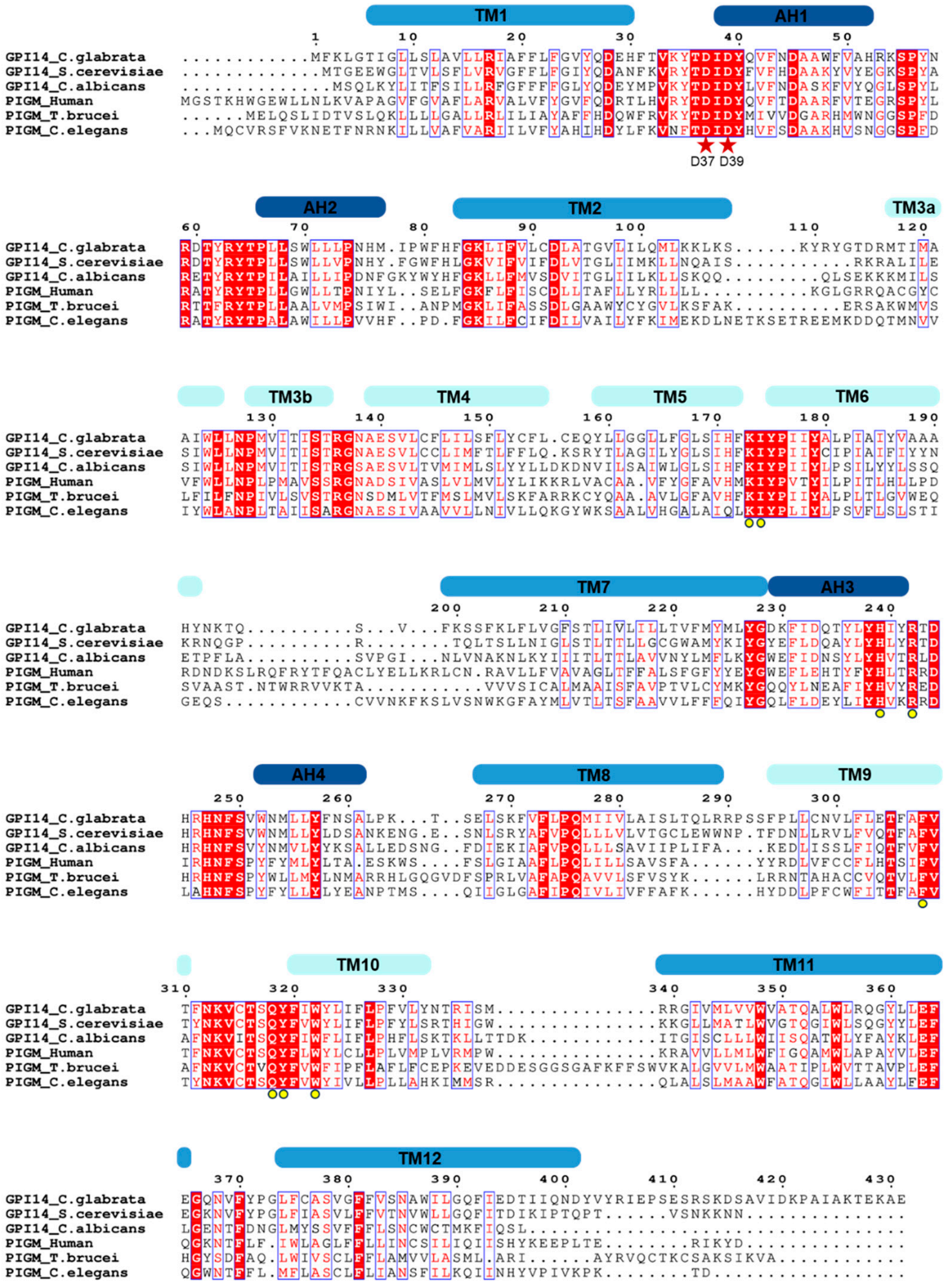

Figure S4. Multiple sequence alignment of Gpi14.

Multiple sequence alignment was performed with Clustal Omega and visualized using ESPript 3. Sequences include Gpi14 from *Candida glabrata* (UniprotKB: Q6FXQ5), *Saccharomyces cerevisiae* (UniprotKB: P47088), *Candida albicans* (UniprotKB: Q5AMR5), and its homolog PIG-M from *Homo sapiens* (UniprotKB: Q9H3S5), *Trypanosoma brucei* (UniprotKB: Q9BPQ5), and *Caenorhabditis elegans* (UniprotKB:

Q17515). The conserved DxD motif is marked by red stars, and residues involved in Dol-P-Man interactions are highlighted with yellow circles.

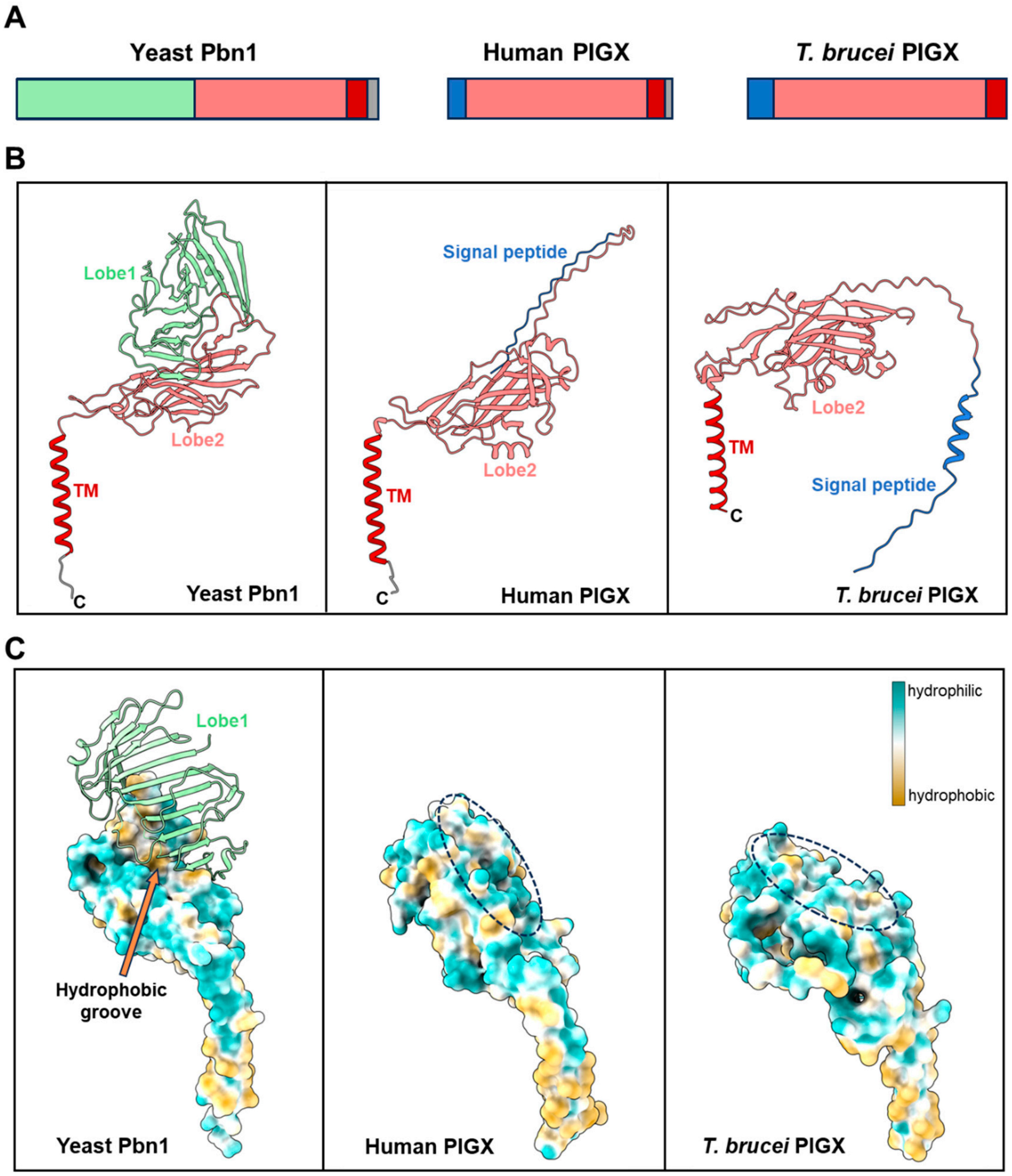

Figure S5. Structural comparison of yeast Pbn1 with its homologs from human and *Trypanosoma brucei*.

(A) Comparison of domain architectures of Pbn1 homologs from yeast, human, and *T. brucei*. Predicted signal peptides, Lobe 1, Lobe 2, the TM helix, and cytoplasmic regions are colored blue, light coral, green, red, and grey, respectively. **(B)** Structural comparison of Pbn1 homologs. Unlike human and *T. brucei* homologs, yeast Pbn1 lacks a signal peptide, with its N-terminus instead forming Lobe 1. **(C)** Hydrophobic surface representation of Pbn1, with Lobe 1 shown in cartoon. The orange arrow indicates how Lobe 2 of yeast Pbn1 encloses Lobe 1 to form a hydrophobic groove, whereas the

regions marked by black dashed circles show that the corresponding interfaces in the human and *T. brucei* homologs are more hydrophilic. Hydrophilic and hydrophobic regions are colored cyan and goldenrod, respectively.

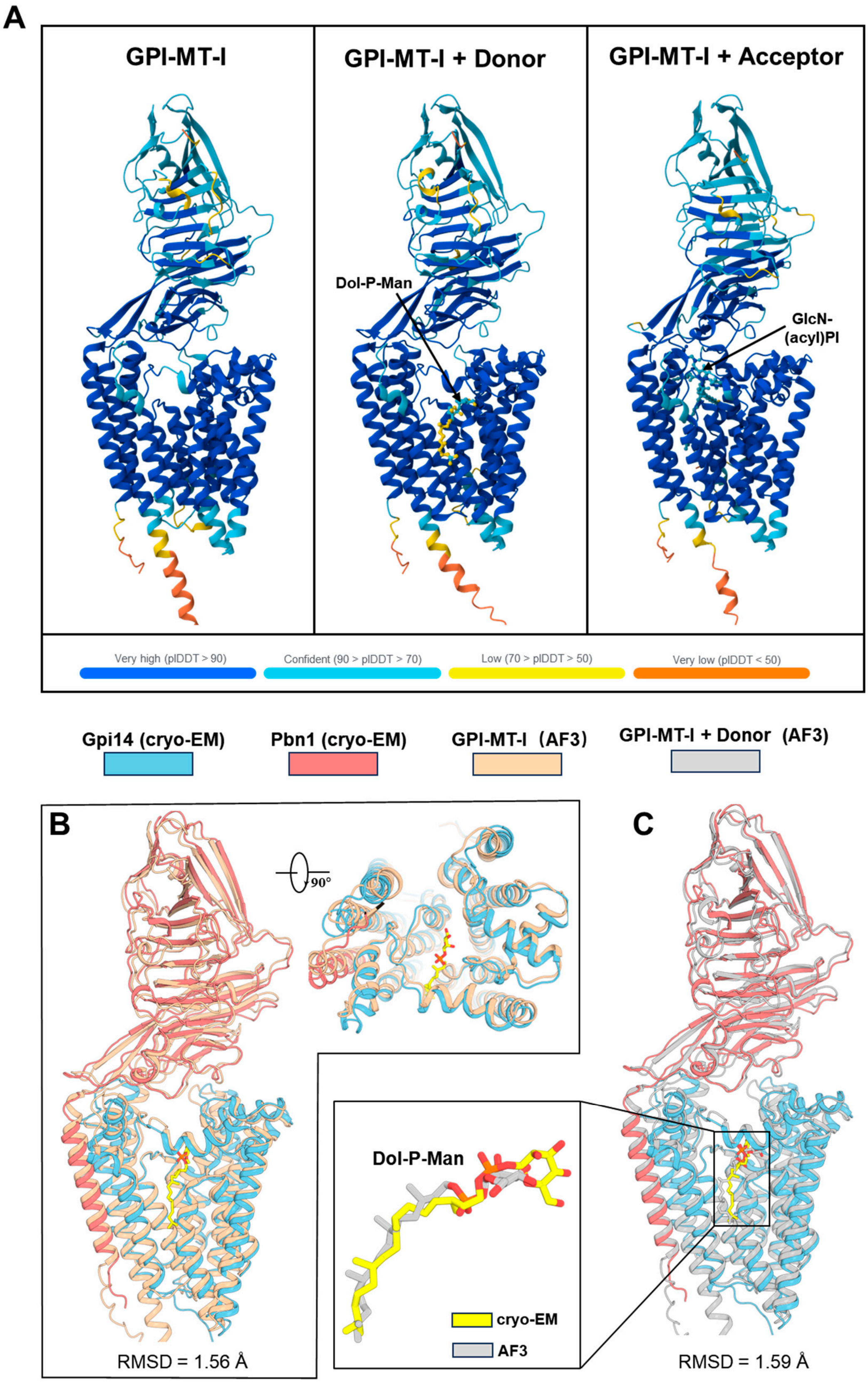

Figure S6. GPI-MT-I structures in different states predicted by AlphaFold3.

(A) Predicted structures of *C. glabrata* GPI-MT-I in the apo state, donor-bound, and

acceptor-bound states. Models are colored by pLDDT scores; ligands are shown as ball-and-stick. **(B)** Side (left) and top (right) views of superposition of the experimentally determined donor-bound structure with the predicted apo state. In the cryo-EM structure, Gpi14 and Pbn1 are colored sky blue and light coral, respectively, while the predicted apo state is shown in wheat. **(C)** Superposition of the experimentally determined donor-bound structure with the AlphaFold3 prediction. The inset highlights differences in Dol-P-Man positioning between the experimental (yellow) and predicted (grey) models.

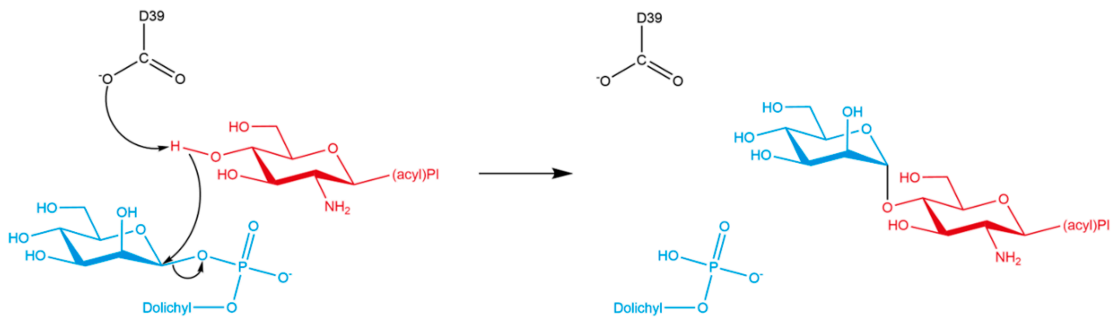

Figure S7. Proposed catalytic mechanism of GPI-MT-I.

Residue D39 functions as the catalytic base, deprotonating the C4 hydroxyl group of the acceptor GlcN. This activates a nucleophilic attack on the phosphate-linked C1 of the donor mannose, resulting in transfer of the mannose to GlcN and formation of an α-1,4 glycosidic bond. Donor and acceptor substrates are colored blue and red, respectively.

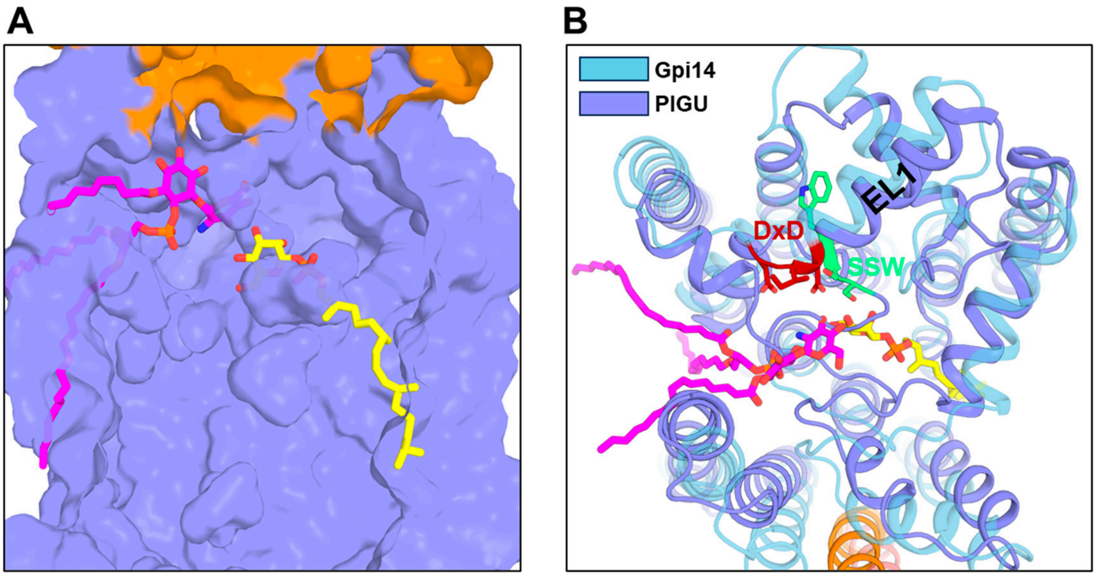

Figure S8. Catalytic channel occlusion in the PIGU–PIGT complex.

(A) Sectional surface view of the PIGU–PIGT complex. The donor (yellow) and acceptor (magenta) from GPI-MT-I are modeled, showing steric clashes with the interior of PIGU–PIGT. **(B)** Superposition of GPI-MT-I and PIGU–PIGT structures. The catalytically essential DxD motif (red) in EL1 of Gpi14 is substituted by an SSW sequence (green) in PIGU.

Table S1. Cryo-EM data collection, refinement, and validation statistics.

	GPI-MT-I (Dol-P-Man)
**Data collection and processing**	
Magnification	81000
Voltage (kV)	300
Electron exposure (e-/Å^2)	~56
Defocus range (μm)	-1.7 to -2.1
Pixel size (Å)	1.088
Symmetry imposed	C1
Final particle images (no.)	80,856
Map resolution (Å)	3.48
FSC threshold	0.143
Map sharpening *B* factor (Å^2)	-152.4
**Refinement**	
R.m.s deviations	
Bond lengths (Å)	0.003
Bond angles (°)	0.574
Validation	
Molprobity score	1.66
Clashscore	6.46
Poor rotamers (%)	0
Ramachandran plot	
Favored (%)	95.56
Allowed (%)	4.44
Disallowed (%)	0.00
